# Vitamin C inhibits crystallization of struvite from artificial urine in the presence of *Pseudomonas aeruginosa*

**DOI:** 10.1590/S1677-5538.IBJU.2017.0656

**Published:** 2018

**Authors:** Muhammed A. P. Manzoor, Surya Ram Duwal, M. Mujeeburahiman, Punchappady-Devasya Rekha

**Affiliations:** 1 Yenepoya Research Centre, Yenepoya Medical College, Yenepoya University, Mangalore, Karnataka, India;; 2 Department of Biochemistry, Yenepoya Medical College, Yenepoya University, Mangalore, Karnataka, India;; 3 Department of Urology, Yenepoya Medical College, Yenepoya University, Mangalore, Karnataka, India

**Keywords:** Struvite, Pseudomonas aeruginosa, Ascorbic Acid

## Abstract

**Background:**

Formation of struvite stones is associated with urinary tract infection by urease-producing bacteria. Biogenic crystal growth in natural and synthetic materials is regulated by the action of inhibitors, ranging from small ions, molecules to large macromolecules.

**Materials and Methods:**

We report the dynamics of *in vitro* crystallization of struvite in presence of vitamin C in synthetic urine using single diffusion gel growth technique. Sodium metasilicate gel of specific gravity 1.05 and the aqueous solution of ammonium dihydrogen phosphate were used as the medium for growing the struvite crystals. The crystallization process was induced by a urease positive struvite stone associated *Pseudomonas aeruginosa* to mimic the infection leading to stone formation. The grown crystals were characterized by ATR-FTIR and powder XRD. The surface morphology was analysed through FE-SEM for comparison between treatments.

**Results:**

We observed decrease in number, dimension, and growth rate of struvite crystals with the increasing concentrations of vitamin C. Crystals displayed well-defined faces and dendritic morphology of struvite in both control and biogenic systems.

**Conclusion:**

The results strongly suggest that, vitamin C can modulate the formation of struvite crystals in the presence of uropathogenic bacteria.

## INTRODUCTION

Struvite or magnesium ammonium phosphate stone is associated with infections by urease-producing bacteria and accounts for about 10-15% of all kidney stones (1). The enzyme urease produced by the bacteria can cause super-saturation and crystallization of Mg^2+^ and PO_4_^3–^ as carbonate apatite (Ca_10_ (PO_4_)_6_.CO_3_) and struvite (MgNH_4_PO_4_.6H_2_O), respectively (2, 3). Struvite crystals can aggregate and form large crystals in the branches of the collecting system, to form large aggregates called staghorns. If untreated, they can cause significant kidney damage, and can sometimes also be life-threatening due to loss of kidney function (4).

Previous study from our group reported the high diversity of mixed stones having two or more than two types of mineral compositions among kidney stone patients (5). Struvite stones can be present as pure types or along with other compositions such as calcium oxalate and hydroxyapatite crystals. Struvite crystallization is mediated by the urease producing bacteria such as: *Staphylococcus* (Gram-positive), *Proteus, Pseudomonas, Providencia* and *Klebsiella* (Gram-negative). In addition, certain species of *Serratia, Corynebacterium* and *Morganella* also produce the enzyme urease which can lead to stone formation. Treatment of struvite stones involves stone removal followed by antibiotic therapy to eliminate bacteria from the urinary tract (6). Crystal growth in biogenic, natural and synthetic medium is regulated by the action of various inhibitors. *In vitro* crystallization of struvite has been increasingly investigated over the last years (7-9). However, bacterially induced struvite crystallization and its inhibition studies have higher implications in healthcare (6, 8, 10).

Compounds such as curcumin and vanillic acid can inhibit the growth of struvite in bacteria--induced crystallization *in vitro* (6, 11). Ascorbic acid, or vitamin C, is an essential micronutrient required for the normal metabolic functions and acts as an electron donor or reducing agent in biochemical reactions (12). Studies on vitamin C in kidney stone disease have shown mixed results with respect to oxalate metabolism and excretion (13, 14). It was initially reported that vitamin C can reduce the urinary pH (15, 16), however, others have found it as an ineffective urinary acidifier (17, 18).

Here we report the role of vitamin C on crystallization and pathogenesis of struvite crystal caused by *P. aeruginosa* isolated from the infectious kidney stone. To best of our knowledge there are no reports on uropathogenic *P. aeruginosa* induced struvite crystallization and inhibition of the same by vitamin C.

## MATERIALS AND METHODS

### 

#### Chemicals and Reagents

Ammonium di-hydrogenphosphate (NH_4_H_2_PO_4_), sodium metasilicate (Na_2_SiO_3_), magnesium acetate solution (C_4_H_6_MgO_4_) and vitamin C were of analytical grade and purchased from commercial sources.

#### Bacterial Strain and Culture Conditions

All the procedures performed in studies involving human participants were approved by the Institutional Scientific Review Board (YRCSRB/034/17) and Institutional Ethics Committee (YUEC.No.2016/022) of the Yenepoya University. *Pseudomonas aeruginosa* strain YU22S, previously isolated from the stone culture of a patient with struvite stone was used. *P. aeruginosa* was tested for urease activity using urea agar and phenol hypochlorite assay (secondary screening). The bacteria were cultured on tryptic soy broth (TSB) for 18h at 37°C and cells were harvested by centrifugation (6000rpm, 8 min). Density of the suspension was determined using McFarland standards and spectrophotometrically (OD 550nm). The cells were suspended in synthetic urine (artificially prepared aqueous solution with mineral compositions for simulating the urine) to an appropriate concentration (10^5^cells/mL).

#### Preparation of Synthetic Urine

The synthetic urine used for the crystallization was prepared according to Griffith et al., (19) (g/L): CaCl_2_ 2H_2_O, (0.651); MgCl_2_ 6H_2_O, (0.651); NaCl, (4.6); Na_2_SO_4_, (2.3); KH_2_PO_4_, (2.8); KCl, (1.6); NH_4_Cl, (1.0); sodium citrate, (0.65); sodium oxalate, (0.02); urea, (25.0); creatine, (1.1); and TSB, (10). The content of the mineral components in the synthetic urine corresponds to mean concentration found in 24h period in normal human urine.

#### Experimental Setup

The single diffusion gel growth technique was used to study the growth and inhibition of struvite crystals as described elsewhere (8, 20). Briefly, sodium metasilicate (SMS) solution of specific gravity 1.05 was used to prepare the gel. An aqueous solution of ammonium di-hydrogenphosphate (0.5M) was mixed with the SMS solution in appropriate amount, so that the gel pH was set at 7.0 in test tubes (140mm length and 25mm diameter). To this, 20mL supernatant solutions of 0.5M magnesium acetate in synthetic urine along with different concentrations of vitamin C were gently poured on the gels without disturbing it. Bacteria suspended in synthetic urine at a density of 5×10^5^ CFU/mL was used for the groups having bacterial addition. All the procedures were done aseptically. Bacterial growth in the synthetic urine was assessed every 24h by plating on trypticase soy agar (TSA) to monitor the viability. All the experiments were performed in triplicates at 37±0.5°C. During the experiment, pH of the samples was measured using a digital pH meter. Additionally, samples from different stages of the crystallization process were observed under a light microscope. Macro-morphology of grown crystals was recorded using stereomicroscope (Carl Zeiss, Göttingen, Germany).

#### Characterization of Struvite Crystals

Samples from different stages of the crystallization process were collected and used for investigations. The grown crystals were harvested from the test tube set up and characterized by Field Emission Scanning Electron Microscope (FE-SEM), Attenuated Total Reflectance-Fourier transform infrared spectroscopy (ATR-FTIR), and X-ray diffraction (XRD). ATR-FTIR of the amorphous crystals was directly recorded using the instrument (Shimadzu IR Prestige-21) and compositions were determined by the FTIR spectra at mid frequency range (4000-400cm^−1^) at 4cm^−1^ resolution. The XRD patterns were recorded with a Rigaku MiniFlex 600 laboratory diffractometer using a Cu-Κα radiation (λ=1.5406Å). Diffraction patterns were registered within the 2° angle range from 10 to 80° and the phase identification was calculated from the diffractograms. The microstructure and morphology were observed using FE-SEM (Carl Zeiss, Germany).

### Statistical analysis

All the experiments were performed in triplicates. Continuous variables are reported as means ± standard deviation. Pearson's correlation was used to correlate the amount of struvite crystals formed in control and test groups. The value of p<0.05 was considered statistically significant. Statistical analysis was performed using SPSS, Version 22.0. (IBM Corp).

## RESULTS

Inhibitory Activity of Vitamin C during Bacteria-induced Struvite Crystallization.

**Figure 1:**
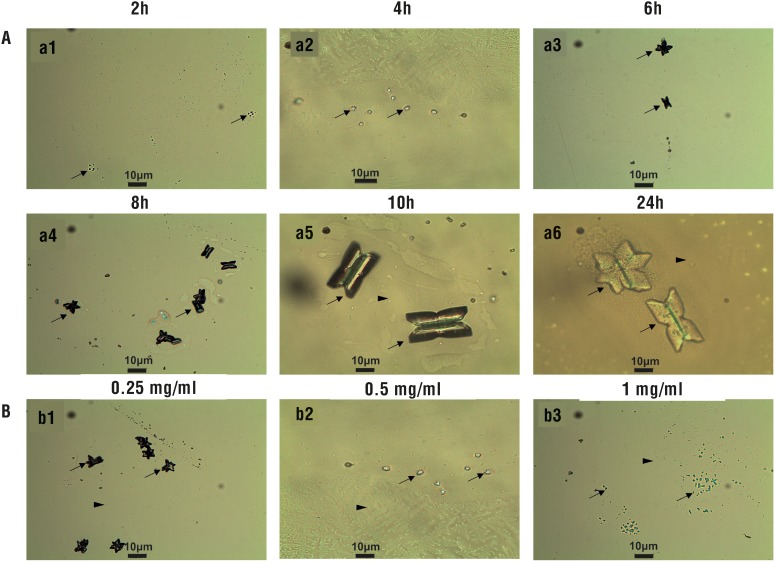
Struvite crystals grown in artificial urine infected with *Pseudomonas aeruginosa* (a). Temporal growth pattern in control (a1-a6) (b). Inhibition of struvite crystals due to vitamin C treatment 24 h (b1:0.25 mg/mL, b2:0.5 mg/mL and b3:1 mg/mL). “Arrow” shows crystals and “arrowhead” shows bacteria.

The early stages (2-24h) of struvite crystallization are given in Figure-1. During this interval, struvite exhibited typical hemimorphic morphology of coffin-lid shape along with bacterial cell. In the presence of different concentrations of vitamin C, crystal formation was delayed compared to control. In both control and vitamin C treated groups, dendritic and X-shaped crystals were formed. However, in the presence of vitamin C, the crystals formed were comparatively smaller and lesser in numbers.

**Figure 2:**
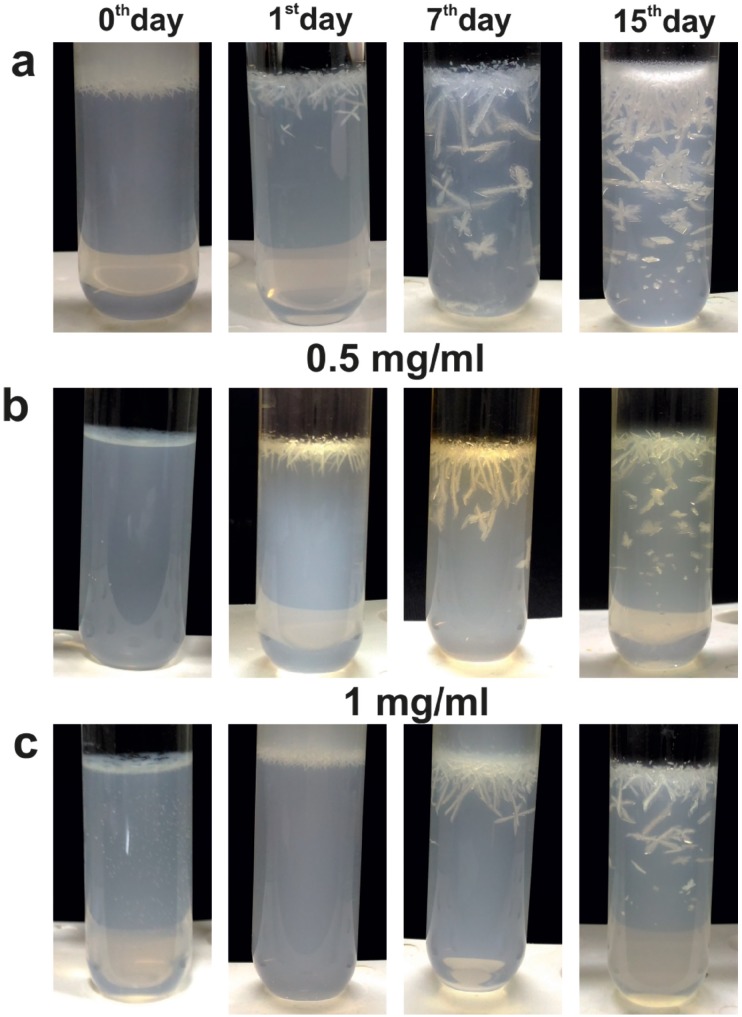
Photograph of the struvite crystals grown in gel medium in artificial urine infected with *Pseudomonas aeruginosa* and treated with different concentrations of vitamin C at different time points (0th, 1st, 7th and 15th day). (a) In the absence of vitamin C (b & c) In the presence of 0.5 mg/mL and 1 mg/mL vitamin C respectively.

Due to the urease activity, at higher pH, the crystals frequently formed twins, and large dendritic branches (Figure-1A, image a6). The time-resolved experiments in the gel media showed the growth of small dendritic type crystals at the end of the first day in the gel at the gel-liquid interface. The initial pinpoint crystals tend to nucleate randomly throughout the test tube and in its surfaces (Figure-2). With the increase in time, the amount of crystals and size gradually increased. However, vitamin C concentrations and the weight of the struvite crystals showed significant inverse correlation (r= 0.962, p<0.05).

### 

#### Effect of pH, Bacterial Viability and Dimensions of Struvite during Crystallization.

**Figure 3:**
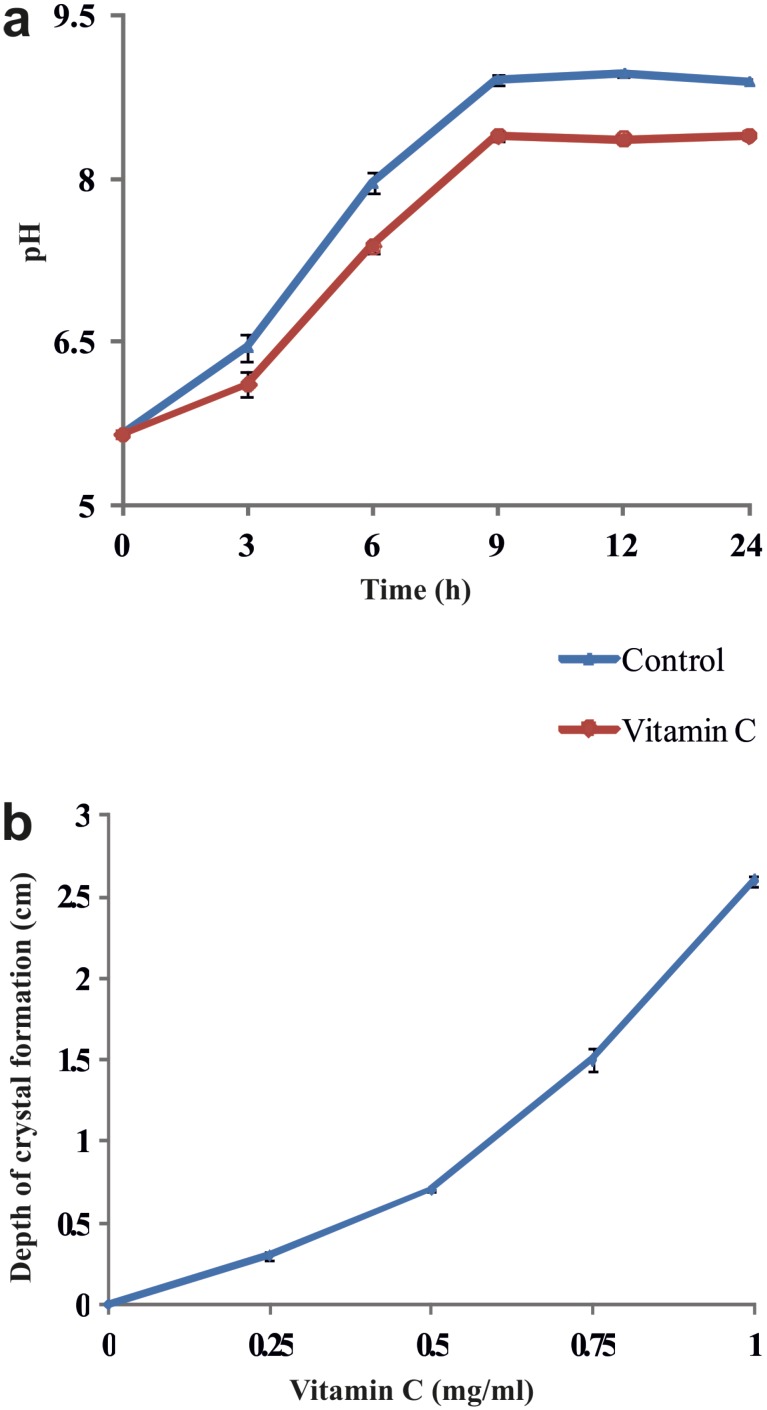
a) Kinetics of pH of the artificial urine infected with *Pseudomonas aeruginosa* without (control) and with vitamin C. b) Depth of struvite crystals formed in the gel media in different concentration of vitamin C.

**Figure 4:**
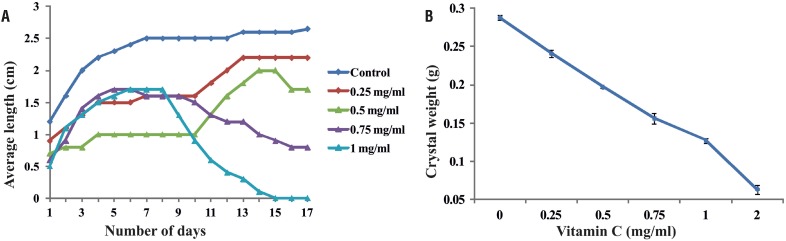
Relationship between vitamin C concentrations and (a) length and (b) weight of struvite crystals obtained in gel growth technique.

In the *P. aeruginosa* infected synthetic urine, a progressive change in the pH was observed during the initial 24h, from pH 5.65 to 8.9. In particular, in the presence of vitamin C, urine pH increased slower compared with the control (Figure-3A). Interestingly, crystal formation presence of vitamin C in the gel was in the higher depth compared to control. This may be possibly due to the penetration of vitamin C into the upper region of the gel. The depth at which crystals formed increased with the concentration of vitamin C (Figure-3B). The size and weight of the harvested crystals showed concentration dependent changes with vitamin C treatment (Figure-4).

#### Structural and Morphological Characterization of Struvite Crystals

**Figure 5:**
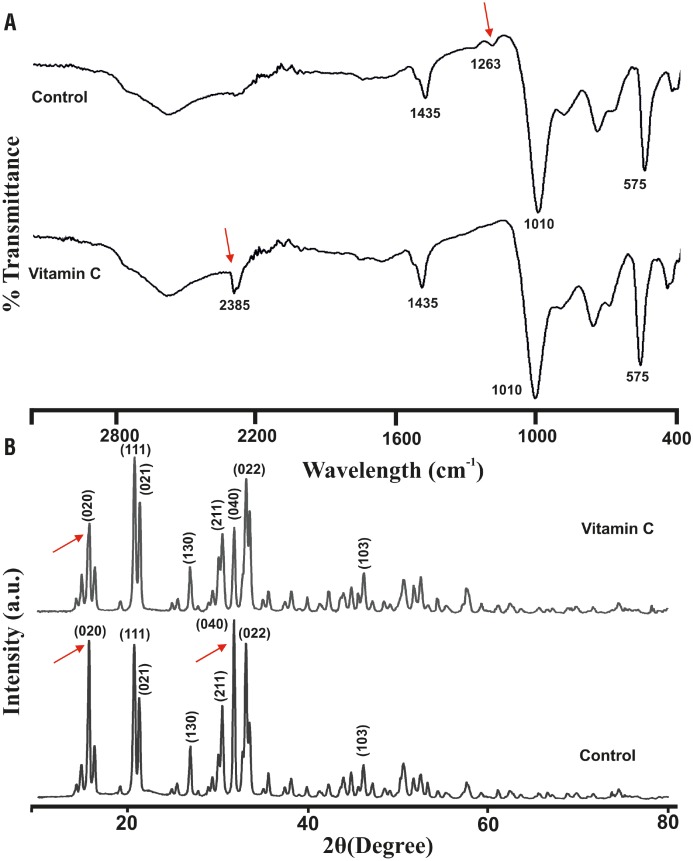
Representative (a) FTIR and (b) XRD patterns of the of the grown struvite crystal. Arrow indicates differences in the peak.

Struvite crystals showed characteristic IR spectrum at 1010cm^−1^ due to the absorption of PO_4_^3–^ (v_3_). Peaks at 1469, 1435 and 1400 cm^−1^ attributed to (v_4_) NH_4_+ bending and the peaks at 892 and 761cm^−1^ correspond to the ammonium-water H bonding and water-water H bonding respectively. The P-O bend (v_4_) and the PO_4_^3–^ (v_2_) modes were represented by peaks at 572 and 462cm^−1^ represent. In the presence of vitamin C, the band at 1263cm^−1^ was absent and a new peak at 2385cm^−1^ was found (Figure-5A). XRD patterns for the crystals obtained with and without vitamin C treatment are shown in Figure-5B. The struvite crystallizes were in the orthorhombic Pmn21 space group (cell parameters a=6.955 Å, b=11.2 Å, c=6.142 Å). Vitamin C induced struvite exhibited increased peak intensity corresponding to (021) and decreased intensity of (020) and (040) planes as compared to the control. It shows that vitamin C interfere in the crystal growth and the preferred growth being at (111) plane. This indicates preferential adsorption and binding of vitamin C onto these faces and results in the prominent development in (111) face.

**Figure 6:**
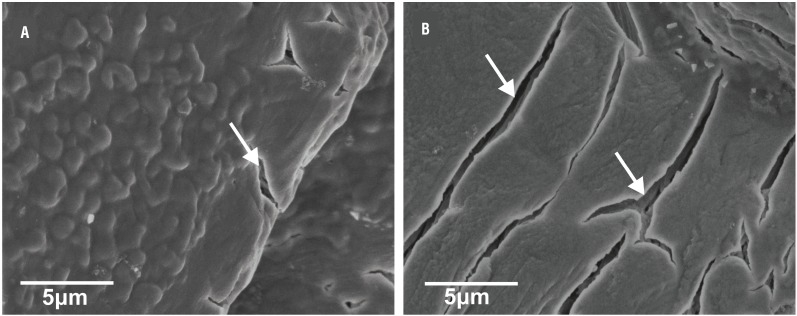
FE-SEM micrographs of struvite crystals grown in artificial urine infected with *Pseudomonas aeruginosa* revealing the porous structure and mesoscopic arrangement (a) control (b) in presence of vitamin C (1 mg/mL). Arrow indicates porous morphology.

The struvite crystals exhibited porous nature with characteristic tubular pores in FE-SEM and the single struvite crystals had well-defined crystalline faces and multi-layered depositions. In addition, vitamin C induced struvite crystals had highly porous appearance compared to control (Figure-6). Moreover, the presence of Mg, N, P and O using X-ray spectroscopy confirmed the major elements present in struvite crystal.

## DISCUSSION

The effect of vitamin C on *P. aeruginosa*-induced struvite crystallization was analysed in detail using microscopy, changes in pH, and bacterial viability during 24h incubation in synthetic urine. Struvite exhibited typical hemimorphic morphology in the presence of uropathogenic bacteria. These characteristic hemimorphic habit and morphology were reported previously in detail (9). And similar observations were also made for struvite crystallized in the presence of *Proteus mirabilis* (8). In the presence of vitamin C, crystal formation was delayed compared to control and X-shaped crystals were observed. These characteristic X-shapes have been reported in bacteria--induced struvite crystallization (8, 21).

The induction time for crystallization in the presence of vitamin C was delayed. Similar observation was also found with other inhibitory compounds during bacteria induced struvite crystallization in artificial urine (11). Moreover, the size and weight of the harvested crystals showed concentration dependent changes with vitamin C treatment. Such inhibitory reduction was observed in struvite crystallization under polyaspartic acid treatment of different concentrations (9). In FTIR spectra, shift in the bands to lower frequencies were seen, which may be due to the vibrations related to stretching Mg-O modes. Such changes in the structure of the struvite crystals were also reported earlier (22, 23). Vitamin C interferes in the crystal growth and the preferred growth being at (111) plane and the XRD pattern of struvite crystals grown in the gel medium are identical with the reported literature (7, 24). The struvite crystals exhibited porous nature and the multi-layered depositions. These multi-layered depositions are commonly observed during the formation of struvite crystals *in vitro* in the presence of natural compounds (24). The internal porous nature with characteristic tubular pores of struvite crystal during *P. mirabilis* induced crystallization was well explained (8).

### Possible Mode of Action for the Struvite Inhibition by Vitamin C

The results obtained with respect to *in vitro* inhibition of struvite showed a decrease of crystal size and weight in the presence of vitamin C in a concentration dependent manner. pH of supernatant solution was acidic in nature, indicating that vitamin C can constantly bring down the pH thereby preventing the crystallization in the synthetic urine. Vitamin C can lower the pH and also inhibit the urease activity due the altered pH. In addition, these characteristics delay the nucleation and appearance of struvite crystals in the presence of vitamin C. Citrus fruits containing vitamin C had similar interaction with struvite minerals and inhibited crystal growth *in vitro* (20).

Blood levels of vitamin C can have a significant role in preventing struvite stone formation. Vitamin C has been shown to play a significant role in preventing infection progression through increased reactive oxygen species production against bacteria (25). Current treatment of infection stones remains challenging, and management of the struvite calculi requires a comprehensive approach. Dietary manipulation, antibiotic therapy along with acidification therapy with vitamin C may improve the clinical outcome of the patients. Furthermore, understanding the mechanism of vitamin C interaction with urinary tract bacteria can be studied to understand its role in the prevention of bacteria-induced struvite. The effect of vitamin C on struvite stone formation needs to be evaluated with other urease-producing bacteria. The effect of vitamin C on struvite stone formation needs to be further evaluated using *in vivo* models to establish the findings of the present study to support vitamin C as a potential choice for prevention of struvite stones.

## CONCLUSIONS

Struvite crystals displayed well-defined faces and dendritic morphology in biogenic systems. Vitamin C has inhibitory activity on bacterially induced struvite crystal formation.

## ABBREVIATIONS

ADP: = Ammonium di-hydrogenphosphate
ATR-FTIR: = Attenuated Total Reflectance-Fourier Transform Infrared Spectroscopy
FE-SEM: = Field Emission Scanning Electron Microscope
SMS: = Sodium metasilicate
TSA: = Trypticase soy agar
TSB: = Tryptic soy broth
XRD: = X-ray diffraction
